# Web-Based Video Platforms as Sources of Information on Body Image Dissatisfaction in Adolescents: Content and Quality Analysis of a Cross-Sectional Study

**DOI:** 10.2196/71652

**Published:** 2025-09-02

**Authors:** Li Liu, Jianning Yang, Fengmei Tan, Huan Luo, Yanhua Chen, Xiaolei Zhao

**Affiliations:** 1 School of Nursing, Southwest Medical University, Luzhou, China; 2 Department of Hematopathology, The Affiliated Hospital, Southwest Medical University, Luzhou, China; 3 Department of Nephrology, The Affiliated Hospital, Southwest Medical University, Luzhou, China; 4 Nursing Department, The Affiliated Hospital, Southwest Medical University, No 1, Section 1, Xianglin Road, Longmatan District, Luzhou, 646000, China, 86 1380250224

**Keywords:** adolescents, body image, video, quality analysis, TikTok, BiliBili, YouTube, GQS, Global Quality Scale, mDISCERN, Modified DISCERN, mJAMA, Modified Journal of the American Medical Association

## Abstract

**Background:**

Body image dissatisfaction among children and adolescents is a significant public health concern and is associated with numerous physical and mental problems. Social media platforms, including TikTok, BiliBili, and YouTube, have become popular sources of health information. However, the quality and reliability of content related to body image dissatisfaction have not been comprehensively evaluated.

**Objective:**

The primary goal of this study was to examine the quality and reliability of videos related to body image dissatisfaction on TikTok, BiliBili, and YouTube.

**Methods:**

The keywords “body image dissatisfaction” were searched on YouTube, TikTok, and BiliBili in November 2024. Videos were collected based on platform-specific sort filters, including the filter of “Most liked” on TikTok and the filter of “Most viewed” on BiliBili and YouTube. The top 100 videos on each platform were reviewed and screened in the study. After excluding videos that were (1) not in English or Chinese, (2) duplicates, (3) irrelevant, (4) no audio or visual, (5) contained advertisements, and (6) with a Global Quality Scale (GQS) score of 1, the final sample consisted of 64 videos, which formed the basis of our research and subsequent findings. Two reviewers (LL and JNY) screened, selected, extracted data, and evaluated all videos using the GQS, the Modified DISCERN (mDISCERN) scores, and the Modified Journal of the American Medical Association (mJAMA) benchmark criteria. Statistical analysis was performed using SPSS (version 28.0; IBM Corp).

**Results:**

In total, 64 videos were analyzed in the study, including 20 from TikTok, 13 from BiliBili, and 31 from YouTube. The median duration of the involved videos was 3.01 (IQR 1.00-5.94) minutes on TikTok, 3.52 (IQR 2.36-5.63) minutes on BiliBili, and 4.86 (IQR 3.10-6.93) minutes on YouTube. Compared with the other 2 platforms, BiliBili videos received higher likes and more comments. The majority of the videos (n=40, 62%) were uploaded by self-media. The quality of the videos on YouTube shows the highest overall scores. Videos uploaded by professional authors had significantly higher GQS, mDISCERN, and mJAMA scores compared to those uploaded by nonprofessionals. There was no significant correlation between video quality and the number of views or likes. However, the number of views and likes were significantly positively correlated. Furthermore, a significant correlation was found between the mJAMA, mDISCERN, and GQS scores.

**Conclusions:**

Web-based video platforms have become an important source for adolescents to access health information. However, the lack of a significant correlation between video quality and the number of likes and comments poses a challenge for users seeking reliable health information. It is suggested that the quality of the videos on health information would be taken into consideration in the recommendation algorithm on web-based video platforms.

## Introduction

Body image is defined as a person’s perceptions, emotions, and thoughts about his or her body [1]. Body image dissatisfaction is the difference between how an individual perceives their physical appearance characteristics and their desire to be [2]. There is a relatively high incidence of body image dissatisfaction among adolescents who undergo psychological and physical changes during puberty and attempt to fit in with cultural expectations of a slim body shape. A web-based international survey with 10,765 youths aged 16 to 25 years from Australia, Canada, New Zealand, the United Kingdom, and the United States showed a body image dissatisfaction rate as high as 75.19% [3]. Another study in Southwest China indicated that 55.2% of young women experienced body image dissatisfaction in a cross-sectional study with 4149 college students in Sichuan province and Chongqing city [4]. Adolescents with body image dissatisfaction are associated with psychological and mental problems such as low self-esteem [5,6], anxiety [7,8], depression [9,10], and chronic dysphoria [11]. It can also lead to a series of serious physical and behavioral issues, such as eating disorders [12], extreme weight loss behaviors [13], alcohol [14], drug abuse [15], excessive pursuit of cosmetic surgeries [16], and suicidality [17,18].

The internet has become an indispensable part of adolescents’ lives [19]. Web-based video platforms have gradually become crucial for adolescents to obtain health information and interact with others [20]. According to a study in Austria, 79% of respondents get their health information through websites [21], which was more prevalent than the doctors. TikTok, BiliBili, and YouTube, because of their ease of use and anonymous accessibility, are preferred for adolescents to obtain health information. Video platforms have become the primary medium for adolescents to seek health information since video content is more vivid and efficient than traditional text-based information. However, it is difficult for users to access the reliability and quality of the video content because of the lack of peer review and guidelines.

Studies examined the quality of educational videos concerning physical diseases on YouTube, covering topics like pain relief [22], kidney transplantation [23], and COVID-19 [24]. It was demonstrated that the quality of the educational videos varied [25]. No studies have analyzed the accuracy and quality of the videos related to body image dissatisfaction among adolescents on web-based video platforms. Therefore, this study aims to assess the accuracy, adequacy, quality, and reliability of the video content on body image dissatisfaction available on TikTok, BiliBili, and YouTube.

## Methods

### Ethical Considerations

This study was conducted without the use of clinical data, human specimens, or laboratory animals. All relevant information was derived from publicly available platforms, including TikTok, BiliBili, and YouTube, and none of the data entailed personal privacy concerns, as they were deidentified. Therefore, an ethics review was deemed unnecessary.

### Data Collection

The keyword “body image dissatisfaction” was searched on YouTube, TikTok, and BiliBili in November 2024. New user accounts were created on each of the 3 web-based video platforms to ensure that the data collected were unbiased, without being influenced by previous search history. Videos were collected based on platform-specific sort filters, including the filter of “Most liked” on TikTok and the filter of “Most viewed” on BiliBili and YouTube. The top 100 videos on each platform were reviewed and screened in the study. The 2 researchers (LL and JNY) reviewed the videos independently. The videos in relation to causes, symptoms, or coping strategies of body image dissatisfaction were included. After excluding videos that were (1) not in English or Chinese, (2) duplicates, (3) no audio or visual, (4) contained advertisements, and (5) with a GQS score of 1, the final sample consisted of 64 videos, which formed the basis of our research and subsequent findings.

The following parameters were documented, including uploaders, upload date, platform, views, likes, comments, shares, saves, video duration, and the quality of the video (Multimedia Appendix 1). However, the following data were unavailable: (1) views on TikTok and (2) shares and saves on YouTube. The uploaders could be divided into individuals (self-media and medical workers), professional organizations, and for-profit corporations, such as Dove. Self-media uploaders were regarded as nonprofessionals, while others were regarded as professionals.

### Video Review

In November 2024, 2 reviewers (LL and JNY) independently reviewed the videos using the 3 quality assessment tools. The videos about causes, symptoms, and coping strategies of body image dissatisfaction were involved. Videos not covering these contents were deemed irrelevant and should be excluded. Discrepancies were resolved through discussion or consultation with the third author (XLZ). A video could have multiple types of content, so the ratios and percentages were reported as higher than the total number of videos.

### Video Quality Assessments

Three widely adopted standardized scales, namely the Global Quality Scale (GQS) [26], Modified DISCERN (mDISCERN) [27], and Modified Journal of the American Medical Association (mJAMA) [28] were selected to assess the quality of the videos.

The GQS is a 5-point Likert-type scale used to measure a video’s overall quality, ﬂow, and usefulness, ranging from 1 to 5 according to quality (a higher score indicates higher quality). Generally, scores of 4‐5 points indicated high quality, 3 points indicated moderate quality, and 1‐2 points indicated low quality [26] (Multimedia Appendix 2).

The mDISCERN, a revised version of the original DISCERN, is more suitable for evaluating video-based content. It consists of 5 standards to assess the reliability of video content, including clarity, relevance, fairness, traceability, and robustness. Each of the above questions was scored 1 point for “yes” and 0 point for “no,” and the cumulative score was calculated as 0‐5 points. Higher scores signified superior reliability and quality (Multimedia Appendix 3).

The mJAMA criteria is a well-known quality assessment tool used to evaluate information from health-related websites. The mJAMA score awards 1 point for the following 4 standards: authorship, attribution, disclosure, and currency. A score of 4 represents the highest quality (Multimedia Appendix 4).

### Statistical Analysis

Statistical analysis was performed using SPSS (version 28.0; IBM Corp). Categorical variables were presented as frequencies and percentages. Continuous variables were expressed as mean and SD or median (IQR). The Shapiro-Wilk tests were used to test the normality of the data distribution. For data with a normal distribution, an independent sample 2-tailed *t* test was used. The Mann-Whitney *U* test was conducted to compare the 2 groups of quantitative data that were not normally distributed. The Kruskal-Wallis test was used to compare 3 or more groups. The Spearman test was conducted to examine the correlation between the scores and video characteristics. The Spearman correlation coefficient was low (ρ<0.40), moderate (ρ=0.40‐0.59), and high (ρ>0.60). Cohen κ was used to measure the agreement for categorical data between the 2 raters, while an intraclass correlation coefficient (ICC) was used to assess interrater reliability on quantitative data. The κ values were interpreted as follows: κ>0.8 indicated outstanding consistency, 0.6<κ≤0.8 suggested good agreement, 0.4<κ≤0.6 signified moderate agreement, and κ≤0.4 was indicative of poor agreement [29]. The ICC values were interpreted as follows: ICC>0.9 indicated outstanding consistency, 0.75<ICC≤0.9 suggested good agreement, 0.5<ICC≤0.75 signified moderate agreement, and ICC≤0.5 was indicative of poor agreement [30]. A 2-tailed *P* value of <.05 indicates statistical significance. For variables with missing data (eg, TikTok views and YouTube shares and saves), listwise deletion was applied.

## Results

### Overview

The ICC values showing the level of agreement between the 2 independent raters (LL and JNY) assessing the videos are presented with 95% CIs. There was excellent statistical agreement among the raters, the ICC for GQS was 0.87 (95% CI 0.78-0.92), the ICC for mDISCERN was 0.84 (95% CI 0.76-0.90), and the ICC for the mJAMA score was 0.86 (95% CI 0.78-0.91), with all *P* values <.001.

### Video Characteristics

**Figure 1.**
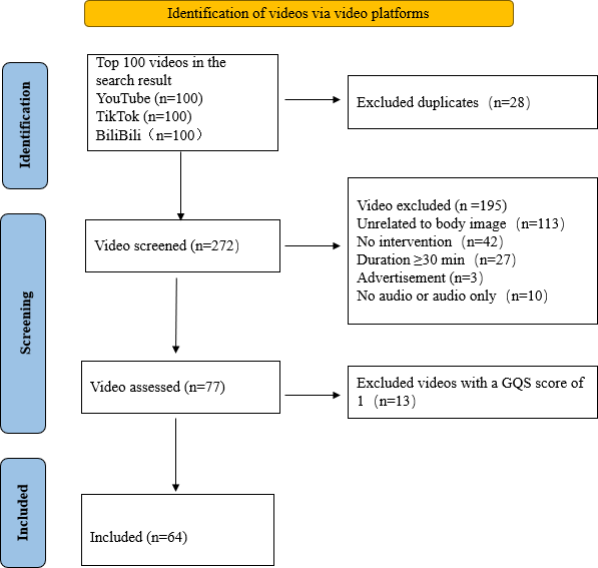
Search strategy for videos on body image dissatisfaction.

This study evaluated 100 videos on 3 platforms, respectively. A flowchart of the video selection process is reported in Figure 1. The 236 videos were excluded due to being duplicates (n=28), having a duration exceeding 30 minutes (n=27), having no audio or audio only (n=10), or being unrelated to body image (n=113). A refined collection of 20 videos from TikTok, 13 from BiliBili, and 31 from YouTube was included for the subsequent analysis. Among the 64 videos included, the uploaders of 40 videos were self-media uploaders, 12 were from professional organizations, 6 were from medical workers, and another 6 were from for-profit corporations. The average duration of the videos included was 4.53 (SD 3.01; range 0.53-14.16) minutes. There were videos with as few as 946 views and those with more than 10,000,000 views. The number of likes varied from 10 to 270,000, with a median of 907 (IQR 10‐270,000). However, views on TikTok and share and save on YouTube are unavailable. The median GQS of these videos was 4 (IQR 2-5), the median mDISCERN score was 3 (IQR 1-5), and the median mJAMA score was 3 (IQR 1-4). For general information about the videos, such as uploaders, likes, time since upload, and duration, see Table 1.

**Table 1.** Detailed characteristics of the videos (n=64).

Parameters	Values, median (IQR)
Duration (minutes)	4.13 (0.53-14.16)
Views^a^	59,000 (946-10,400,000)
Likes	907 (10‐270,000)
Shares^b^	475 (1‐86,000)
Saves^b^	341 (4‐75,000)
Times since video upload (months)	28.5 (1-142)
GQS^c^ score	4 (2-5)
mDISCERN^d^ score	3 (1-5)
mJAMA^e^ score	3 (1-4)

a Data from BiliBili and YouTube.

b Data from TikTok and BiliBili.

c GQS: Global Quality Scale.

d mDISCERN: Modified DISCERN.

e mJAMA: Modified Journal of the American Medical Association.

### Video Content Analysis

Content analysis was conducted in the involved videos. Coping strategies were the most frequently discussed topics, with 84% (n=54) of the videos providing suggestions for self-acceptance, refusal to make comparisons, social support seeking, and life adjustment. Concerning the treatments of body image dissatisfaction, cognitive behavioral therapy and meditation are the most widely applied, as shown in Textbox 1. In addition, causes and symptoms of body image dissatisfaction were also topics of public concern, with 23% (n=15) and 52% (n=33) of videos providing full explanations, respectively.

Textbox 1. **Recommended therapies and tips for body image dissatisfaction.**

**Ten recommended therapies for body image dissatisfaction**

1. Cognitive behavioral therapy (n=5)
2. Meditation (n=5)
3. Selective serotonin reuptake inhibitors (n=3)
4. Mirror therapy (n=2)
5. Sports participation (n=2)
6. Mindfulness (n=1)
7. Dance therapy (n=1)
8. Hypnotherapy (n=1)
9. Talk therapy (n=1)
10. Point percussion therapy (n=1)

**Ten tips for building a positive body image**

1. Self-acceptance (n=14)
2. Social support seeking (n=8)
3. Stay away from negative comments (n=7)
4. Focus on your strengths (n=6)
5. Express gratitude to your body (n=6)
6. Stop comparing (n=5)
7. Define beauty by yourself (n=3)
8. Positive self-affirmation (n=2)
9. Take part in social activities (n=2)
10. Wear clothes that make you feel good and truly suit you (n=1)

### Characteristics Comparison of Different Platforms

The characteristics and the quality of the involved videos on 3 web-based video platforms are presented in Table 2. The median duration of the included video sources was 3.01 (IQR 0.53‐8.75) minutes on TikTok, which was shorter than that on BiliBili (median 3.52, IQR 1.18‐14.16 minutes) and YouTube (median 4.86, IQR 1‐13.15 minutes). There was a significant difference in video duration between TikTok and YouTube. The median time since the included videos uploaded was 18 (IQR 1-38) months on TikTok, which was significantly shorter than those of the videos posted on BiliBili (median 30, IQR 13-91 months) and YouTube (median 35, IQR 3-142 months). For the video characteristics, there were no statistics differences in views, likes, comments, or saves among the 3 groups. However, the median number of likes and comments was 2169 (IQR 39‐193,000) and 60 (IQR 3‐2527) on BiliBili, respectively, which were higher than that on TikTok and YouTube. The median number of views on YouTube was higher than those videos on BiliBili, and the median number of shares and saves on BiliBili was higher than those videos on TikTok. In terms of video quality, the GQS, mDISCERN, and mJAMA scores of the included videos on YouTube were significantly better than those of videos posted on TikTok and BiliBili.

**Table 2.** Characteristics of video platforms about body image dissatisfaction.

Characteristic	Platforms			*P* value^a^		
	TikTok (n=20), median (IQR)	BiliBili (n=13), median (IQR)	YouTube (n=31), median (IQR)	*P*_T-B_ ^b^	*P*_B-Y_ ^c^	*P*_T-Y_ ^d^
Time since video upload (months)	18 (1‐38)	30 (13‐91)	35 (3‐142)	.04^e^	.10	<.001^e^
View	—^f^	29,000 (946‐1,323,000)	117,437 (2020‐10,400,000)	—	.29	—
Likes	537 (13‐238,000)	2169 (39‐193,000)	1502 (0‐270,000)	.54	.92	.58
Comments	37.5 (0‐15,000)	60 (3‐2527)	36 (0‐16,634)	.47	.76	.99
Share	396 (1‐86,000)	475 (34‐23,000)	—	.84	—	—
Save	237 (4‐39,000)	718 (24‐75,000)	—	.11	—	—
Duration (minutes)	3.01 (0.53‐8.75)	3.52 (1.18‐14.16)	4.86 (1‐13.15)	.31	.28	.03^e^
GQS^g^	3 (2‐4)	3.0 (2‐5)	4 (2‐5)	.98	.02^e^	.01^e^
mDISCERN^h^	2.5 (1‐4)	2 (1‐5)	4 (2‐5)	.50	<.001^e^	<.001^e^
mJAMA^i^	2 (2‐3)	2.0 (1‐3)	4 (2‐4)	.52	<.001^e^	<.001^e^

a All the *P* values were obtained from Mann-Whitney *U* test.

b *P*_T-B_: TikTok versus BiliBili.

c *P*_B-Y_: BiliBili versus YouTube.

d *P*_T-Y_: TikTok versus YouTube.

e *P* value <.05.

f Not available.

g GQS: Global Quality Scale.

h mDISCERN: Modified DISCERN.

i mJAMA: Modified Journal of the American Medical Association.

### Characteristics Comparison of Different Video Sources

Table 3 shows the characteristics of video uploaders about body image dissatisfaction on the 3 web-based video platforms. The result showed that most videos (n=40, 63%) were uploaded by self-media. The median duration of the videos posted by medical workers was 6.56 (IQR 2.53‐13.15) minutes, which was longer than the videos from other video sources. The median number of likes was 2313 (IQR 10‐35,000) for videos posted by professional organizations, which was higher than the videos posted by self-media uploaders (median 1384, IQR 13‐27,000), medical workers (median 1204, IQR 0‐5611), and commercial corporations (median 456, IQR 50‐81,000). The median number of comments was 49.5 (IQR 0‐16,634) for videos posted by self-medias, which was more than those videos from other video sources. Regarding the video quality, videos uploaded by professionals had significantly higher GQS, mDISCERN, and mJAMA scores compared with those uploaded by nonprofessionals.

**Table 3.** Characteristics of video uploaders about body image dissatisfaction on TikTok or BiliBili or YouTube.

Characteristics	Uploaders				*P* value					
	Self-medias (n=40), median (IQR)	Professional organizations (n=12), median (IQR)	Medical workers (n=6), median (IQR)	For-profit corporations (n=6), median (IQR)	*P*_s-p_ ^a^	*P*_p-m_ ^b^	*P*_m-c_ ^c^	*P*_s-c_ ^d^	*P*_s-m_ ^e^	*P*_p-c^f^_
Time since video upload (months)	26 (3‐142)	30 (1‐110)	38.5 (31‐95)	24 (3‐64)	.62	.20	.09	.55	.03^g^	.38
Views	—^h^	—	34,500 (8858‐190,000)	203,409 (15,971‐3,087,713)	—	—	.24	—	—	—
Likes	1384 (13‐27,000)	2313 (10‐35,000)	1204 (0‐5611)	456 (50‐81,000)	.98	.49	.99	.57	.47	.55
Comments	49.5 (0‐16,634)	37.5 (0‐2334)	37 (0‐667)	0 (0‐415)	.68	.82	.13	.01^g^	.64	.08
Duration (minutes)	4.13 (0.53‐14.16)	4.05 (1.67‐7.68)	6.56 (2.53‐13.15)	3.29 (1‐7.36)	.82	.10	.13	.81	.09	.89
GQS^i^	3.5 (2‐5)	3 (3‐4)	4 (2‐5)	4 (3‐5)	.99	.49	.93	.31	.49	.29
mDISCERN^k^	3 (1‐5)	4 (1‐5)	4 (3‐5)	4.5 (4‐5)	.03^g^	.49	.48	.01^g^	.08	.18
mJAMA^j^	2 (1‐4)	4 (2‐4)	4 (3‐4)	4 (4‐4)	<.001^g^	.75	.39	<.001^g^	<.001^g^	.18

a *P*_s-p_: self-medias versus professional organizations.

b *P*_p-m_: professional organizations versus medical workers.

c *P*_m-c_: medical workers versus corporations.

d *P*_s-c_: self-medias versus corporations.

e *P*_s-m_: self-medias versus medical workers.

f *P*_p-c_: professional organizations versus corporations.

g *P* value <.05.

h Not available.

i GQS: Global Quality Scale.

j mJAMA: Modified Journal of the American Medical Association.

k mDISCERN: Modified DISCERN.

### Correlation Analysis

A correlation analysis was performed between different video characteristics and different scores in Table 4. A strong correlation was found between the uploaders and platforms (*r*=0.641; *P*<.001), as well as a moderate correlation between platforms and the time since videos were uploaded (*r*=0.497; *P*<.001), and a low correlation between platforms and video duration (*r*=0.28; *P*=.02). Moderate to strong correlations were observed between views and likes (*r*=0.880; *P*<.001) and views and comments (*r*=0.561; *P*<.001). A strong correlation was found between mDISCERN and mJAMA (*r*=0.67; *P<*.001), a moderate correlation between GQS and mDISCERN (*r*=0.558; *P*<.001), and a weak correlation between GQS and mJAMA (*r*=0.306; *P*=.01). Additionally, low to strong correlations were found between platforms and mJAMA (*r*=0.782; *P*<.001), mDISCERN (*r*=0.594; *P*<.001), and GQS (*r*=0.39; *P<*.001). Low to moderate correlations were observed between the time since videos were uploaded and mDISCERN (*r*=0.454; *P*<.001), GQS (*r*=0.405; *P*<.001), and mJAMA (*r*=0.368; *P*<.001).

**Table 4.** Correlation analyses^a^.

		Uploaders	Time since video upload	Platform	Views	Likes	Comments	Duration	GQS^b^	mDISCERN^c^	mJAMA^d^
Uploaders											
	*r*	—^e^	0.106	0.641	0.097	0.092	−0.23	0.007	0.133	0.518	0.777
	*P* value	—	.41	<.001^f^	.54	.47	.07	.58	.29	<.001^f^	<.001^f^
Time since video upload											
	*r*	0.106	—	0.497	0.186	0.11	0.188	0.175	0.405	0.454	0.369
	*P* value	.41	—	<.001^f^	.23	.38	.14	.17	<.001^f^	<.001^f^	<.001^f^
Platform											
	*r*	0.641	0.497	—	0.192	0.072	0.02	0.282	0.393	0.594	0.782
	*P* value	<.001^f^	<.001^f^	—	.21	.57	.99	.02^f^	<.001^f^	<.001^f^	<.001^f^
Views											
	*r*	0.097	0.186	0.192	—	0.880	0.561	0.011	−0.033	0.128	0.201
	*P* value	.54	.23	.21	—	<.001^f^	<.001^f^	.94	.83	.41	.19
Likes											
	*r*	0.092	0.11	0.072	0.880	—	0.778	−0.084	−0.056	0.007	0.030
	*P* value	.47	.38	.57	<.001^f^	—	<.001^f^	.51	.66	.95	.81
Comments											
	*r*	−0.23	0.188	0.02	0.561	0.778	—	0.089	−0.048	−0.048	−0.079
	*P* value	.07	.14	.99	<.001^f^	<.001^f^	—	.49	.71	.71	.54
Duration											
	*r*	0.07	0.175	0.282	0.011	−0.084	0.089	—	0.463	0.317	0.201
	*P* value	.58	.17	.02^f^	.94	.51	.49	—	<.001^f^	.01^f^	.11
GQS											
	*r*	0.133	0.405	0.393	−0.033	−0.056	−0.048	0.463	—	0.558	0.306
	*P* value	.29	<.001^f^	<.001^f^	.83	.66	.71	<.001^f^	—	<.001^f^	.01^f^
mDISCERN											
	*r*	0.518	0.454	0.594	0.128	0.007	−0.048	0.317	0.558	—	0.674
	*P* value	<.001^f^	<.001^f^	<.001^f^	.41	.95	.70	.01^f^	<.001^f^	—	<.001^f^
mJAMA											
	*r*	0.777	0.369	0.782	0.201	0.030	−0.079	0.204	0.306	0.674	—
	*P* value	<.001^f^	<.001^f^	<.001^f^	.19	.81	.54	.11	.01^f^	<.001^f^	—

a All the *P* values were obtained from Spearman ρ.

b GQS: Global Quality Scale.

c mDISCERN: Modified DISCERN.

d mJAMA: Modified Journal of the American Medical Association.

e Not available.

f *P* value <.05.

## Discussion

### Principal Findings

Web-based video platforms provide unprecedented opportunities for viewers to swiftly obtain and disseminate health information [31], including allowing health communication for adolescents with body image dissatisfaction. The length of the videos on TikTok was shorter than that of the videos on BiliBili and YouTube (*P*=.03), and the time since videos were uploaded was shortest on TikTok. The audience engagement metrics (median likes or comments) were highest on BiliBili. The majority of videos (n=40, 63%) were uploaded from self-media. Videos uploaded by medical workers, organizations, and corporations demonstrated significantly higher GQS, mDISCERN, and mJAMA scores compared with those uploaded by self-media. An important finding was the lack of significant correlation between the objective video quality metrics (GQS, mDISCERN, and mJAMA) and user engagement indicators (likes and comments), indicating that the popularity of videos would not reflect their quality.

This study found a moderate correlation between platforms and the time since videos were uploaded. The median time since videos were uploaded was 18 (IQR 1-38) months on TikTok, which was significantly shorter than those of BiliBili (median 30, IQR 13-91 months) and YouTube (median 35, IQR 3-142 months), consistent with previous research [32]. This may reflect that platform algorithms prioritize novelty on TikTok. The study also showed that BiliBili had higher likes and comments than TikTok and YouTube. This could be attributed to BiliBili’s unique features, such as bullet-screen comments, which may enhance interactivity among viewers. However, differences in audience demographics (eg, age and cultural background) and uploader popularity could also contribute to engagement disparities. Additionally, among the included videos, BiliBili focused more on storytelling. These findings align with the narrative transportation theory [33], in which storytelling facilitates emotional immersion and reduces counterarguing, thereby enhancing perceived authenticity and trust [34]. This may explain why videos uploaded by professionals, despite higher quality scores, received fewer comments. Besides, there was no correlation between video quality and audience engagement in digital literacy, and users may prioritize emotional resonance over source credibility in this study. This highlights an urgent need for platforms to integrate trust cues, such as verified author badges and quality ratings, into interfaces to guide users to access reliable videos [35].

### Comparison to Prior Work

In terms of video quality, the videos about body image dissatisfaction on YouTube were significantly better than those on TikTok and BiliBili. This result was inconsistent with previous research that mentioned the quality was better on BiliBili [36]. In this study, over 93% (n=31) of the videos on TikTok and BiliBili were uploaded by self-media with more poor content about body image dissatisfaction. On YouTube, a considerable proportion of the videos was uploaded by medical workers and profit corporations. According to the mDISCERN and mJAMA scores, videos uploaded by professionals were significantly better than those uploaded by nonprofessionals, which is consistent with the results of previous studies [37,38]. Additionally, the included videos from profit corporations received fewer comments. This may be because these videos were mostly from Dove with high-quality animation productions, but these videos were silent animations lack of textual explanations.

Low-to-moderate positive correlations were observed between the time since the videos were uploaded and the scores on mDISCERN, GQS, and mJAMA, indicating that higher scores on these assessments may take a considerable amount of time to develop. The video quality score of BiliBili and YouTube was better than TikTok because they promote deeper engagement through in-depth discussion, which requires a longer time to be fully presented and a relatively longer production cycle. Low to strong correlations were found between GQS, mDISCERN, and mJAMA scores. This is consistent with results reported in prior studies [39], indicating the mutual verifiability among video quality assessment tools. There was no significant correlation between video quality and the number of likes or comments. This result, consistent with previous similar studies [29], may indicate that viewers often do not care about the quality of health-related videos.

### Strengths and Limitations

#### Strengths

The quality assessment using GQS, mDISCERN, and mJAMA scales provided comprehensive reliability. Cross-platform comparison (TikTok, BiliBili, and YouTube) in English and Chinese improved the generalizability of the result. The interrater reliability with κ value of 0.767 indicated a good agreement between the raters.

#### Limitations

This study has several limitations. First, the sample size was relatively small, with only 64 videos included, which could not represent all the videos about body image dissatisfaction on the 3 video platforms. Second, the videos were restricted to Chinese and English, which would overlook potentially valuable body image dissatisfaction videos in other languages. This also may lead to the omission of important information about body image dissatisfaction in different cultural backgrounds. Third, this study only analyzed the latest 100 videos in the search results, and some high-quality videos uploaded previously may have been missed. Fourth, the unavailability of TikTok views and YouTube shares and saves may introduce bias in correlation analysis. Fifth, the study did not account for potential confounders such as audience demographics, uploader popularity when exploring the causes of differences in video engagement, and quality. In the future, the researchers will focus on the development of high-quality body image dissatisfaction videos by following scientific processes. It is suggested that the quality of the videos on health information would be taken into consideration in the recommendation algorithm.

### Future Directions

Future research should include videos with more languages, cultures, and video types. Longitudinal analysis tracking video quality and engagement over time is needed to understand how algorithms impact content quality. Platforms can integrate quality criteria into algorithms by collaborating with medical experts. A specific bullet could be designed by the platforms to indicate the quality of the videos by using GQS, mDISCERN, and mJAMA scales.

### Conclusions

This study offers a valuable exploration into the landscape of videos regarding body image dissatisfaction on TikTok, BiliBili, and YouTube. We discovered that web-based video platforms have indeed emerged as significant sources of health information for the public. However, the lack of a significant correlation between video quality and the number of likes and comments poses a challenge for users seeking reliable health information. The quality of the videos on health information would be taken into consideration in the recommendation algorithm on web-based video platforms in the future.

## Supplementary material

10.2196/71652 Multimedia Appendix 1 Parameters.

10.2196/71652 Multimedia Appendix 2 Global Quality Scale.

10.2196/71652 Multimedia Appendix 3 Modified DISCERN.

10.2196/71652 Multimedia Appendix 4 Modified Journal of the American Medical Association.

## Abbreviations

GQS: Global Quality Scale
ICC: intraclass correlation coefficient
mDISCERN: modified DISCERN
mJAMA: Modified Journal of the American Medical Association
