# Supplementary material for: Web-Based Video Platforms as Sources of Information on Body Image Dissatisfaction in Adolescents: Content and Quality Analysis of a Cross-Sectional Study
Source: JMIR Form Res. 2025 Sep 2;9:e71652. doi: 10.2196/71652 (PMC12439227; doi:10.2196/71652)
Supplement: Multimedia Appendix 2 [file formative-v9-e71652-s002.docx]

|  | GQS |
| --- | --- |
| Score | Description |
| 1 | Poor quality, poor flow, most information missing, not useful for education. |
| 2 | Generally poor quality and flow, of limited use to patients because only some information is present but many important topics missing. |
| 3 | Moderate quality, suboptimal flow, somewhat useful for patients as some important information is adequately discussed but others poorly discussed |
| 4 | Good quality, generally good flow, useful to patients because most relevant information is covered but some topics not covered. |
| 5 | Excellent quality and flow, highly useful to patients |
