# Supplementary material for: Web-Based Video Platforms as Sources of Information on Body Image Dissatisfaction in Adolescents: Content and Quality Analysis of a Cross-Sectional Study
Source: JMIR Form Res. 2025 Sep 2;9:e71652. doi: 10.2196/71652 (PMC12439227; doi:10.2196/71652)
Supplement: Multimedia Appendix 3 [file formative-v9-e71652-s003.docx]

| Modified DISCERN |
| --- |
| 1. Is the aim clear, concise, and understandable? |
| 2. Are sources of information reliable? (Cited publication, video content was from valid studies, dentists, and endodontists); |
| 3. Is the information presented balanced and unbiased? (Any reference to other treatment choices) |
| 4. Are additional sources of information listed? |
| 5. Does the video address areas of uncertainty? |
