# Supplementary material for: Web-Based Video Platforms as Sources of Information on Body Image Dissatisfaction in Adolescents: Content and Quality Analysis of a Cross-Sectional Study
Source: JMIR Form Res. 2025 Sep 2;9:e71652. doi: 10.2196/71652 (PMC12439227; doi:10.2196/71652)
Supplement: Multimedia Appendix 4 [file formative-v9-e71652-s004.docx]

| mJAMA | | | |
| --- | --- | --- | --- |
| Authorship | Authors and contributors, their affiliations, and relevant credentials should be provided | Yes=1 | No=0 |
| Attribution | References and sources for all content should be listed clearly, and all relevant copyright information should be noted | Yes=1 | No=0 |
| Disclosure | Ownership” should be prominently and fully disclosed, as should any sponsorship, advertising, underwriting, commercial funding arrangements or support, or potential conflicts of interest | Yes=1 | No=0 |
| Currency | Dates when content was posted and updated should be indicated | Yes=1 | No=0 |
